# Target occupancy study and whole-body dosimetry with a MAGL PET ligand [^11^C]PF-06809247 in non-human primates

**DOI:** 10.1186/s13550-022-00882-2

**Published:** 2022-03-04

**Authors:** Ryosuke Arakawa, Akihiro Takano, Sangram Nag, Zhisheng Jia, Nahid Amini, Kevin P. Maresca, Lei Zhang, Edmund J. Keliher, Christopher R. Butler, Justin R. Piro, Tarek A. Samad, Deborah Smith, Deane Nason, Steve O’Neil, Patrick Trapa, Kari R. Fonseca, John Litchfield, Timothy McCarthy, Richard E. Carson, Christer Halldin

**Affiliations:** 1 Centre for Psychiatry Research, Department of Clinical Neuroscience, Karolinska Institutet, Karolinska University Hospital Solna, R5:02, 17176 Stockholm, Sweden; 2 Stockholm Health Care Services, Stockholm County Council, Stockholm, Sweden; 3 Worldwide Research and Development, Pfizer Inc., Cambridge, MA USA; 4 Department of Radiology and Biomedical Imaging, Yale University School of Medicine, New Haven, CT USA

**Keywords:** MAGL, Non-human primate, Occupancy, PET, Radiation dose

## Abstract

**Background:**

Monoacylglycerol lipase (MAGL) is a key serine hydrolase which terminates endocannabinoid signaling and regulates arachidonic acid driven inflammatory responses within the central nervous system. To develop [^11^C]PF-06809247 into a clinically usable MAGL positron emission tomography (PET) radioligand, we assessed the occupancy of MAGL by an inhibitor in the non-human primate (NHP) brain. Additionally, we measured the whole-body distribution of [^11^C]PF-06809247 in NHP and estimated human effective radiation doses.

**Methods:**

Seven cynomolgus monkeys were enrolled for brain PET measurements. Two PET measurements along with arterial blood sampling were performed in each NHP: one baseline and one pretreatment condition with intravenous administration of PF-06818883, a pro-drug of a selective MAGL inhibitor (total of seven doses between 0.01 and 1.27 mg/kg). Kinetic parameters *K*_1_, *k*_2_ and *k*_3_ were estimated by a two tissue compartment (2TC) model using metabolite corrected plasma radioactivity as the input function. *k*_4_ was set as 0 according to the irreversible binding of [^11^C]PF-06809247. *K*_*i*_ by 2TC and Patlak analysis were calculated as the influx constant. The target occupancy was calculated using *K*_*i*_ at baseline and pretreatment conditions. Two cynomolgus monkeys were enrolled for whole-body PET measurements. Estimates of the absorbed radiation dose in humans were calculated with OLINDA/EXM 1.1 using the adult male reference model.

**Results:**

Radioactivity retention was decreased in all brain regions following pretreatment with PF-06818883. Occupancy was measured as 25.4–100.5% in a dose dependent manner. Whole-body PET showed high radioactivity uptake values in the liver, small intestine, kidney, and brain. The effective dose of [^11^C]PF-06809247 was calculated as 4.3 μSv/MBq.

**Conclusions:**

[^11^C]PF-06809247 is a promising PET ligand for further studies of MAGL in the human brain.

**Supplementary Information:**

The online version contains supplementary material available at 10.1186/s13550-022-00882-2.

## Background

Monoacylglycerol lipase (MAGL) is a serine hydrolase highly expressed throughout the brain and is responsible for converting 2-arachidonylglycerol (2-AG) to arachidonic acid (AA) [1, 2]. As such, the MAGL hydrolytic activity has an important role in the regulation of endocannabinoid signaling as well as inflammatory responses. Inhibition of MAGL has been proposed as a therapeutic strategy for a variety of central nervous system (CNS) injuries and disorders including status epilepticus [3], dysfunction of blood brain barrier permeability [4] and neurodegenerative diseases such as Alzheimer’s disease, Parkinson’s disease and multiple sclerosis, as well as neuropsychiatric disorders. [5–7]. Although a development of MAGL inhibitors is still at a pre-clinical stage, quantitative evaluations in in vivo human brain are necessary for clinical application of these drugs in the future.

Positron emission tomography (PET) is a useful modality for evaluating the proof of concept of novel drugs. Favorable PET radioligands require suitable properties such as high brain accumulation and high specificity for the target protein. Rationale design of a glycol-derived MAGL PET radioligand was accomplished as PF-06809247 (IC_50_ = 13 nM), which was successfully radio-labelled by [^11^C]methylation ([^11^C]PF-06809247) in high molar activity [8]. The preliminary evaluation showed high uptake in non-human primate (NHP) brain, and clear blocking effect by a MAGL inhibitor.

To further develop [^11^C]PF-06809247 into a clinically usable PET radioligand, we assessed the relationship between MAGL target occupancy in the NHP brain and the plasma exposure for a MAGL inhibitor. Additionally, we measured the whole-body distribution of [^11^C]PF-06809247 in NHP and estimated the human effective radiation doses based on the NHP data.

## Methods

The study was approved by the Animal Ethics Committee of the Swedish Animal Welfare Agency (N185/14) and was performed according to “Guidelines for planning, conducting and documenting experimental research” (Dnr 4820/06-600) of Karolinska Institutet. The NHPs were housed in the Astrid Fagraeus Laboratory of the Swedish Institute for Infectious Disease Control, Solna, Sweden.

### Radioligand synthesis

[^11^C]PF-06809247 was synthesized as reported previously [8] (Additional file 1). The precursor and reference standard of radioligand were provided from Pfizer Inc.

### Brain PET measurements

Seven cynomolgus monkeys (two females and five males, body weight 5850–8000 g) were used. Anesthesia was induced by intramuscular injection of ketamine hydrochloride (10 mg/kg) at Astrid Fagraeus Laboratory and maintained by the administration of a mixture of isoflurane (1.5–2.0%), oxygen and medical air through endotracheal intubation. The subjects’ cranium was immobilized with a fixation device. Body temperature was maintained by a Bair Hugger model 505 warming unit (Arizant Healthcare, MN) and monitored by an esophageal thermometer. Heart rate, blood pressure, respiratory rate and oxygen saturation were continuously monitored throughout the experiments. Fluid balance was maintained by continuous infusion of saline.

PET measurements were conducted using a High Resolution Research Tomograph (HRRT) (Siemens Molecular Imaging). A transmission scan of 6 min using a single ^137^Cs source was performed before the [^11^C]PF-06809247 injection. List mode data were acquired continuously for 123 min (first two NHPs) or 63 min (remaining five NHPs) immediately after intravenous injection of the radioligand. Images were reconstructed with a series of 34 frames (20 s × 9, 1 min × 3, 3 min × 5, and 6 min × 17) for 123 min data or 33 frames (10 s × 9, 15 s × 2, 20 s × 3, 30 s × 4, 1 min × 4, 3 min × 4, and 6 min × 7) for 63 min data. The ordinary Poisson-3D-ordered subset expectation maximization (OP-3D-OSEM) algorithm was applied with 10 iterations and 16 subsets including modeling of the point spread function (PSF) [9]. Two PET measurements per NHP were performed in 1 day: one baseline and one following pretreatment.

### MRI measurements

T1-weighted magnetic resonance (MR) images of the individual NHP brains had been obtained using a 1.5 T GE Healthcare Signa system (GE, Milwaukee, Wis, USA). A spoiled gradient recalled (SPGR) sequence had been acquired in the coronal plane with the following parameters: TR = 21 ms; TE = 4 ms; flip angle = 35°; Slice thickness = 1.0 mm; FOV = 12.8 cm; NEX = 2; voxel size = 0.5 × 0.5 × 1 mm^3^.

### Arterial blood sampling

An automated blood-sampling system (ABSS) was used to continuously measure the radioactivity for the first 3 min after the radioligand injection. Blood sampling was performed manually for the measurement of radiometabolism and radioactivity at 2 (only for radiometabolism), 4, 10, 20, 30, 60 (also 90 and 120 for first two NHPs) min after the injection.

### Radiometabolite analysis

A reversed-phase radio-HPLC method was used to determine the amount of unchanged [^11^C]PF-06809247 and its radioactive metabolites in NHP plasma [10]. The plasma obtained after centrifugation of blood at 2000*g* for 2–4 min was mixed with acetonitrile. The mixture was then centrifuged at 2000*g* for 2–4 min and the extract was injected into a HPLC system coupled to an on-line radioactivity detector. The radio-HPLC system used consisted of an interface module (D-7000; Hitachi: Tokyo, Japan), a L-7100 pump (Hitachi), an injector (model 7125, with a 5.0-mL loop; Rheodyne: Cotati, USA), and an ultraviolet absorption detector (L-7400, 254 nm; Hitachi) in series with a 150TR; Packard (housed in a shield of 50 mm thick lead) equipped with a 550 μL flow cell. Chromatographic separation was achieved on a XBridge C18 column, (50 mm × 10 mm I.D., 2.5 μm + 10 mm × 10 mm I.D., 5 μm; Waters: New England, USA) by gradient elution. Acetonitrile (A) and 20 mM ammonium phosphate (pH 7) (B) were used as the mobile phase at 6.0 mL/min, according to the following program: 0–3.5 min, (A/B) 20:80 → 55:45 v/v; 3.5–4.0 min, (A/B) 55:45 v/v; 4.0–4.1 min, (A/B) 55:45 → 20:80 v/v; 4.1–5.0 min, (A/B) 20:80 v/v. Peaks for radioactive compounds eluting from the column were integrated and their areas were expressed as a percentage of the sum of the areas of all detected radioactive compounds (decay-corrected to the time of injection on the HPLC).

### Protein binding

A blood sample was taken at 3 min before injection for measurement of protein binding and determination of free fraction of [^11^C]PF-06809247 in the plasma. The free fraction, *f*_p_, of [^11^C]PF-06809247 in plasma was estimated using an ultrafiltration method [11]. Plasma (400 µL) or phosphate buffered saline solution (400 µL) as a control were mixed with [^11^C]PF-06809247 (40 µL, ~ 1 MBq) and incubated at room temperature for 10 min. After the incubation, 200 µL portions of the incubation mixtures were pipetted into ultrafiltration tubes (Centrifree YM-30, molecular weight cutoff, 30,000; Millipore: Billerica, USA) and centrifuged at 1500*g* for 15 min. Equal aliquots (20 µL) of the ultrafiltrate (*C*_free_) and of the plasma (*C*_total_) were counted for their radioactivity with a 2480 Wizard2 Automatic Gamma Counter (Perkin Elmer: Massachusetts, USA). Each determination was performed in duplicate. The free fraction was then calculated as *f*_p_ = *C*_free_/*C*_total_, and the results were corrected for the membrane binding measured with the control samples.

### Drug administration

PF-06818883 was administered intravenously as a bolus infusion (15 s, volume; 0.5 mL/kg) of seven different doses (0.01–1.27 mg/kg) approximately 1 h before PET scanning [12] (Table 1). PF-06818883 was formulated by dissolving in PBS with a final pH of 7.5. A single dose was administered to four NHPs while three NHPs received two different doses.

**Table 1:** Dose of PF-06818883, plasma concentration of PF-06807893, and MAGL occupancy

	Dose (mg/kg)	Conc (ng/mL)	2TC *K*_*i*_ (%)	Patlak slope (%)
NHP1	1.27	338.5	94.1	99.4
NHP2	1.27	370.0	90.8	100.5
NHP3	0.03	0.7	32.0	34.8
	0.14	10.3	91.5	99.3
NHP4	0.03	2.2	59.9	60.4
	0.42	45.6	94.1	98.8
NHP5	0.07	10.3	86.3	90.9
	0.01	–	22.8	25.4
NHP6	0.14	9.8	91.1	96.4
NHP7	0.055	1.4	47.8	49.9

*NHP* non-huma primate, *Conc* concentration of plasma, *2TC* two-tissue compartment model

### Measurement of plasma concentration of PF-06807893

Venous blood samples (1 mL each) were taken at − 63, − 30, − 1, 30, 60 (also 90 and 120 for first two NHPs) min after the radioligand injection of PET measurements to measure the plasma concentration of PF-06807893. PF-06818883 is a pro-drug and it converts to PF-06807893, an irreversible MAGL inhibitor (IC_50_ = 7 nM). The blood samples were collected in a plasma-tube containing K2 EDTA as an anticoagulant. The plasma samples were harvested by centrifuging the blood sample at a speed of 1200×*g* for 10 min at 4 °C. The harvested plasma samples were immediately stored in − 80 °C freezer. The plasma concentration of PF-06807893 was measured at an analysis laboratory (Unilabs York Bioanalytical Solutions, UK).

### Brain image analysis

The regions of interest (ROIs) were delineated manually on MRI images of each NHP for the whole brain, cerebellum, caudate, putamen, thalamus, frontal cortex, temporal cortex, and hippocampus. The summed PET images of the whole duration were co-registered to the MRI image of the individual NHP. After applying the co-registration parameters to the dynamic PET data, the time-activity curves of brain regions were generated for each PET measurement.

### Kinetic model analysis

Kinetic parameters as *K*_1_, *k*_2_ and *k*_3_ were estimated by two tissue compartment (2TC) using metabolite corrected plasma radioactivity as the input function [13]. *K*_1_ and *k*_2_ are uptake and clearance rate constants between arterial plasma and non-displaceable compartment. *k*_3_ is a rate constant from non-displaceable to specific binding compartment. *k*_4_, a rate constant from specific binding to non-displaceable compartment, was set as 0 according to the irreversible binding of [^11^C]PF-06809247. As the main outcome measures, *K*_*i*_ defined as (*K*_1_ × *k*_3_)/(*k*_2_ + *k*_3_) and Patlak slope were calculated [14]. *K*_*i*_ represents an uptake rate constant that incorporates both net inward transport and trapping in the tissue. Due to low reliable data for parent fraction of [^11^C]PF-06809247 at later phase, only up to 30 min data was used for the quantification. The relation between K_i_ by 2TC and Patlak slope was evaluated by linear correlation.

### Estimation of the target occupancy

The target occupancy was calculated by the following equation: Occupancy (%) = (*K*_*i*_baseline _− *K*_*i*_pretreatment_)/*K*_*i*_baseline_ × 100, as *K*_*i*_baseline_ is *K*_*i*_ at baseline condition and *K*_*i*_pretreatment_ is *K*_*i*_ at pretreatment condition. The *K*_*i*_ values calculated by 2TC and Patlak slope were used to determine the occupancy. The average occupancy by all ROIs was used for further evaluation.

The relationship between average plasma concentration (*C*_ave_) of − 1 and 30 min of PF-06807893 (active metabolite) and occupancy using Patlak slope was estimated by an *E*_max_ model with the following equation: Occupancy (%) = *C*/(EC_50_ + *C*) × E_max_, as *C* is the plasma concentration of PF-06807893, EC_50_ is the plasma concentration required to achieve 50% of the maximum occupancy, and *E*_max_ is maximum occupancy. In this analysis, *E*_max_ was set as 100%.

### Whole-body PET measurements

Whole-body PET measurements were made in two cynomolgus monkeys (two females, body weight 5350 and 5600 g). Anesthesia was administered by intramuscular injection of ketamine hydrochloride (approximately 10 mg/kg) at AFL and maintained by intravenous infusion of ketamine (4 mg/kg/h) and xylazine (0.4 mg/kg/h). The body of the NHP was immobilized using a vacuum pad. Body temperature was maintained by a Bair Hugger model 505 and monitored by an esophageal thermometer. Heart rate, blood pressure, and oxygen saturation were continuously monitored throughout the experiments. Fluid balance was maintained by continuous infusion of saline.

Whole-body PET scans were conducted using a GE Discovery PET/CT 710 (GE healthcare, Waukesha, WI, USA) with around 5 mm full width half maximum (FWHM). One low-dose CT scan was performed before intravenous administration of [^11^C]PF-06809247 for attenuation correction. Then four series of PET acquisitions, each covering four axial fields of view (AFOV), were conducted. The four PET series consisted of two 20 s × 4 AFOV scans, three 40 s × 4 AFOV scans, four 80 s × 4 AFOV scans, and six 160 s × 4 AFOV scans respectively. PET images were reconstructed with a 3D ordered-subset expectation maximization (OSEM) algorithm with three iterations and eighteen subsets, including the time of flight information (VUE Point FX) and the point spread function correction (Sharp IR). A 2D Gaussian filter with 5.5 mm cut-off was used. The time for the bed to return to the original position was approximately 20 s, and the total duration of the whole-body scan was 100 min.

### Image analysis of the whole-body PET

Regions of interest (ROIs) were drawn on the brain, heart, liver, kidney, lung, stomach, spleen, bone (lumbar vertebrae), gall bladder, urinary bladder and small intestine with the help of the CT images for anatomic landmarks. Radioactivity concentration in each PET scan was decay-corrected to the time of injection. Time activity curve was expressed as percentage of the injected dose (%ID) calculated as follows: radioactivity (Bq/cc) × ROI volume (cc)/injected dose (Bq) × 100.

### Radiation dose estimation

Estimates of the absorbed radiation dose for humans was calculated with OLINDA/EXM 1.1 (Organ Level INternal Dose Assessment code) software, using the adult male (70 kg) reference model [15]. The fractional uptake in NHP organs was assumed to be equal to the uptake in human organs.

## Results

### Brain PET

**Fig. 1:**
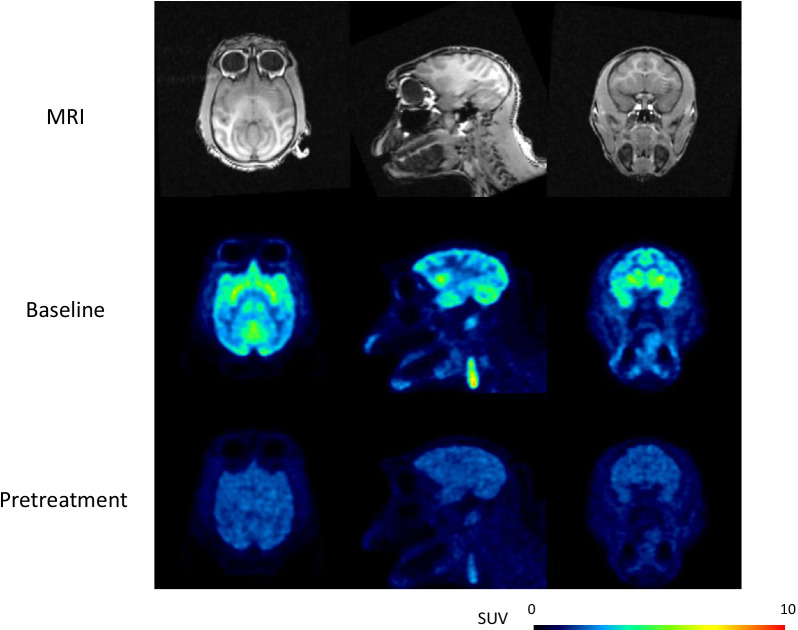
Representative MRI and PET summation images (0–63 min) of [^11^C]PF-06809247 at baseline and following pretreatment with a selective MAGL inhibitor (NHP4; 0.42 mg/kg)

**Fig. 2:**
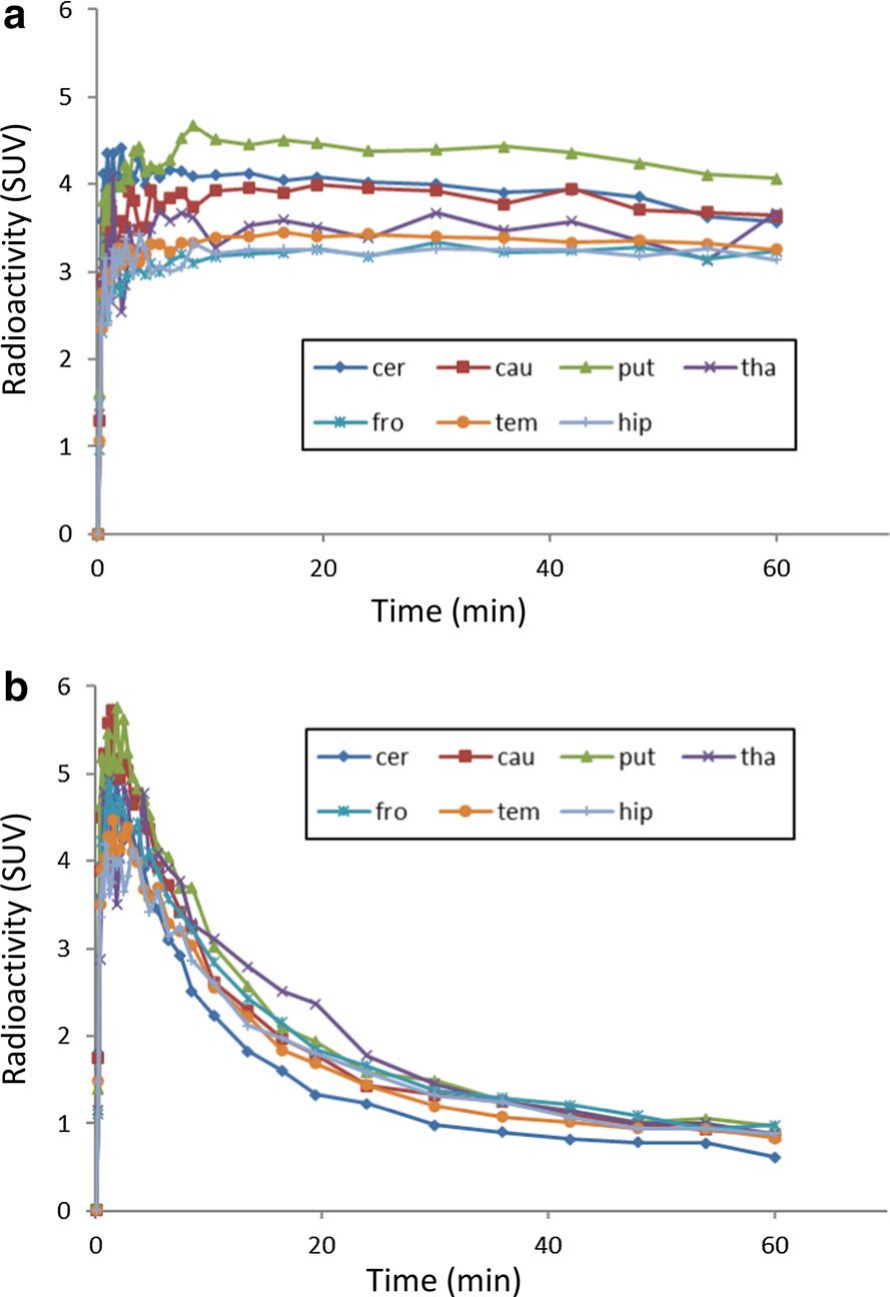
Representative time activity curves of [^11^C]PF-06809247 in the NHP brain at **a** baseline and **b** following pretreatment with a selective MAGL inhibitor (NHP4; 0.42 mg/kg). *cer* cerebellum, *cau* caudate, *put* putamen, *tha* thalamus, *fro* frontal cortex, *tem* temporal cortex, *hip* hippocampus

The injected radioactivity (*n* = 20) of [^11^C]PF-06809247 was 146 ± 10 (mean ± SD) (range; 120–162) MBq. The molar radioactivity at the time of injection was 1510 ± 963 (392–4388) GBq/µmol, and the injected mass was 0.06 ± 0.04 (0.01–0.15) µg. Retention of brain uptake was clearly decreased after pretreatment of PF-06818883 (NHP4; 0.42 mg/kg, Fig. 1). TACs for several brain regions demonstrated decreased radioactivity, which corresponded to PET images (NHP4; 0.42 mg/kg, Fig. 2a, b, Additional file 1: Figure S1).

**Fig. 3:**
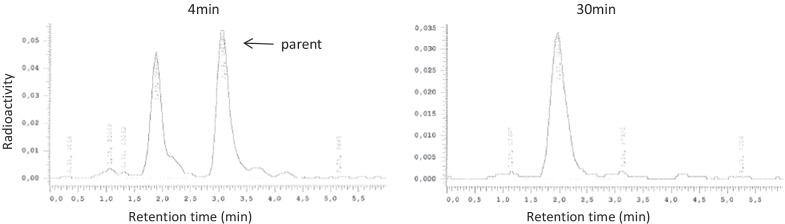
Radio-HPLC of baseline condition at 4- and 30-min post administration of [^11^C]PF-06809247

All radiometabolites showed shorter retention times than the parent (Fig. 3). The percent values of plasma parent fraction became less than 10% after 30 min. The percent values of plasma protein binding were 86.1 ± 2.8%.

**Fig. 4:**
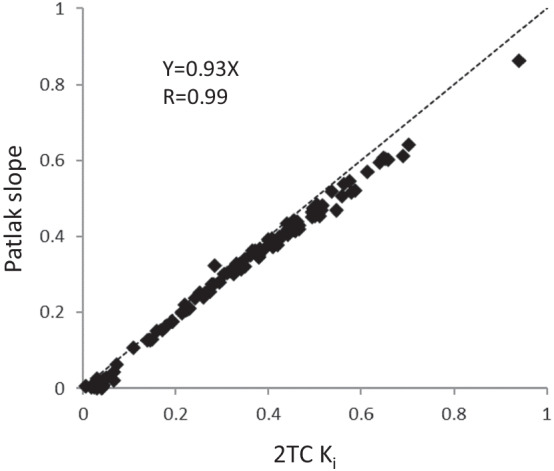
Correlation of *K*_*i*_ measured by 2TC model and Patlak slope

**Fig. 5:**
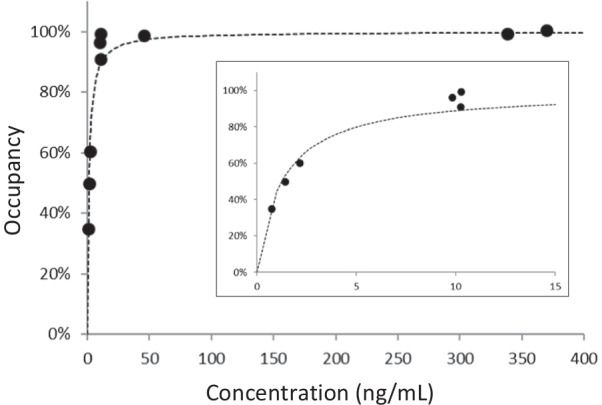
Relationship between plasma concentration of PF-06807893 and MAGL occupancy

*K*_*i*_ by 2TC and Patlak slope were well correlated (Table 2 and Fig. 4). The occupancy range was calculated as 22.8–94.1% and 25.4–100.5% by 2TC *K*_*i*_ and Patlak slope, respectively (Table 1). The average plasma concentration at − 1 and 30 min of PF-06807893 was 0.7–370.0 ng/mL. (Additional file 1: Figure S2) One plasma concentration (dose of 0.01 mg/kg for NHP5) showed below lower limit of detection. The pro-drug PF-06818883 was not detected during the PET measurement in most cases (data not shown). EC_50_ of PF-06807893 was estimated to be 1.3 ng/mL (Fig. 5).

**Table 2:** Kinetic parameters and *K*_*i*_ by 2TC and Patlak slope

	*K*_1_ (ml/ccm/min)		*k*_2_ (1/min)		*k*_3_ (1/min)		2TC *K*_*i*_ (ml/ccm/min)		Patlak slope (ml/ccm/min)	
	Mean	SD	Mean	SD	Mean	SD	Mean	SD	Mean	SD
cer	0.849	0.361	0.645	0.392	0.750	0.373	0.461	0.142	0.425	0.119
cau	0.710	0.313	0.655	0.535	1.335	0.871	0.471	0.110	0.444	0.098
put	0.899	0.547	0.780	0.731	1.287	0.524	0.550	0.166	0.512	0.147
tha	0.643	0.215	0.441	0.344	0.824	0.444	0.430	0.092	0.413	0.073
fro	0.498	0.177	0.265	0.235	0.883	0.640	0.388	0.096	0.371	0.090
tem	0.551	0.220	0.308	0.282	0.782	0.483	0.403	0.098	0.384	0.089
hip	0.621	0.179	0.475	0.413	0.717	0.437	0.393	0.082	0.371	0.075

*cer* cerebellum, *cau* caudate, *put* putamen, *tha* thalamus, *fro* frontal cortex, *tem* temporal cortex, *hip* hippocampus, *SD* standard deviation, *2TC* two-tissue compartment model

### Whole body PET

**Fig. 6:**
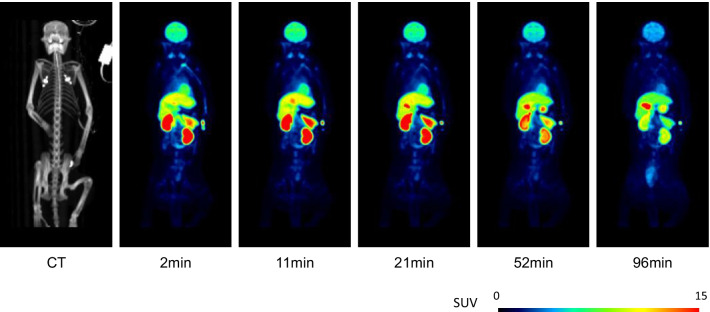
Representative whole-body PET images of [^11^C]PF-06809247

**Fig. 7:**
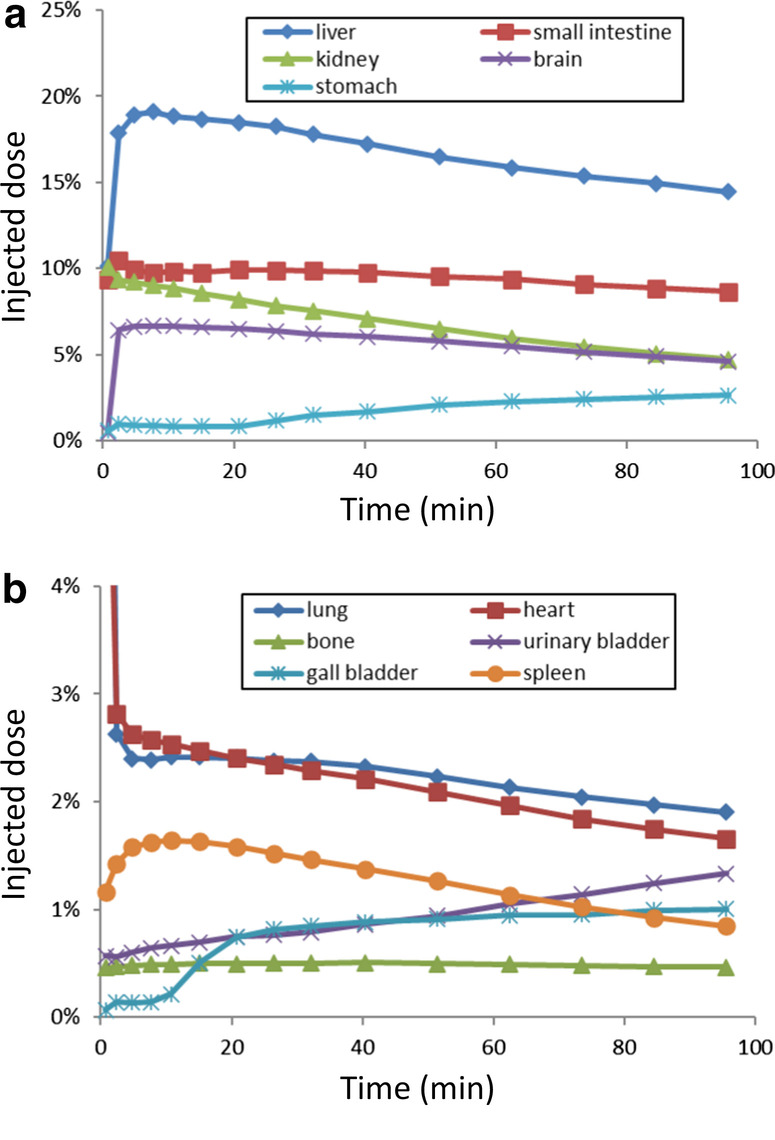
Representative time activity curves of [^11^C]PF-06809247 in **a** high accumulation organs and **b** low accumulation organs

The injected radioactivity of [^11^C]PF-06809247 was 272 and 302 MBq for the two measurements, respectively. The molar radioactivity at the time of injection was 186 and 494 GBq/µmol, and the injected mass was 0.6 and 0.3 µg for the two measurements, respectively. High uptakes were observed in the liver, small intestine, kidney, and brain (Figs. 6, 7a, b). The effective dose is 4.3 μSv/MBq (Table 3).

**Table 3:** The radiation dose estimates of [^11^C]PF-06809247

Target organ	(µSv/MBq)
Adrenals	3.5
Brain	6.9
Breasts	1.7
Gallbladder wall	4.3
LLI wall	2.8
Small intestine	17.2
Stomach wall	2.7
ULI wall	7.4
Heart wall	10.8
Kidneys	39.7
Liver	12.6
Lungs	3.8
Muscle	2.0
Ovaries	3.3
Pancreas	3.5
Red marrow	2.2
Osteogenic cells	2.7
Skin	1.5
Spleen	10.3
Testes	1.6
Thymus	2.0
Thyroid	1.8
Urinary bladder wall	5.3
Uterus	3.1
Total body	2.8
Effective dose	4.3

## Discussion

In this study, we present a novel MAGL PET radioligand, [^11^C]PF-06809247 which showed high uptake in the NHP brain and clear blocking effect by a selective MAGL inhibitor. The uptake of [^11^C]PF-06809247 was relativley uniform thoughout the brain with slightly higher uptake in the celleberum and putamen and lower uptake in the cerebral cortical regions. The distribution was relatively similar as MAGL mRNA expression in the rat brain previously reported [16]. Brain uptake was clearly decreased in all brain regions following pretreatment with a high dose of a previously described selective MAGL inhibitor. The relationship between plasma concentration and occupancy was observed in a dose-dependent manner. This suggests that [^11^C]PF-06809247 is sensitive enough to detect a range of MAGL inhibition and demonstrates the ammenability of [^11^C]PF-06809247 for assessing MAGL occupancy in vivo.

The peak brain uptake was higher at a pretreatment condition than baseline although the decreasing was rapid. Compared to baseline condition, the peak radioactivity of the plasma input function after pretreatment was around 1.8 times higher on average. (Additional file 1: Figure S3) That was one possible explanation for increasing brain uptake. It might be due to peripheral blocking by pretreatment drug although there was no data about whole body imaging of pretreatment condition.

The radio-HPLC data showed that [^11^C]PF-06809247 was rapidly metabolized to less than 10% of total radioactivity in plasma by 30 min. The major radiometabolite could be separated by radio-HPLC and had a retention time that was short (1.9 min) compared to parent fraction (3.1 min), indicating a low probability of brain penetration by the radiometabolite. The metabolisms of PET radioligands among the species such as rodent, NHP and human often show large variabilities. This factor affects the kinetics in the brain according to the change of plasma input function [17]. Despite relatively similar metabolism between human and NHP, actual human studies are needed for accurate evaluations. Additionally, the metabolism of PET radioligand in the brain should be considered. Because of the lack of available data for this issue, further evaluetion also will be needed.

The 2TC model with 3-parameters using arterial input function could describe the TACs of [^11^C]PF-06809247. A 2TC model with 4-parameters did not descirbe the data well (data not shown). Also, the ratio of brain uptake to metabolite-corrected plasma showed continuously increasing at the duration of quantification (Additional file 1: Figure S4). These imply that [^11^C]PF-06809247 has irreversible binding properties and is indeed consistent with prior pharmacological study [8]. The Patlak method was also well fitted (Additional file 1: Figure S5), and the Patlak slope was well correlated with *K*_*i*_ by 2TC model estimation although a slight underestimation (around 7%) was observed. This suggests that graphical analysis would be useful for further evaluations such as parametric image analysis.

The whole-body measurement showed highest accumulation in the liver and small intestine, and relatively low accumulation in the urinary bladder. Notably, the uptake in the brain was also high (7% ID at peak). The effective dose of [^11^C]PF-06809247 was 4.3 μSv/MBq, which was similar to the median value (4.7 μSv/MBq) of other carbon-11 PET radioligands [18]. This radiation exposure would allow multiple administrations in a single subject enabling longitudinal and/or baseline blocking studies in human.

Recently, several studies for NHP brain imaging using PET radioligands for MAGL were reported using [^11^C]MAGL-0519 [19], [^11^C]SAR127303 [20], [^11^C]MA-PB-1 [21], [^18^F]T-401 [22], and [^18^F]PF-06795071 [1]. In addition, a report highlighting the opportunities and challenges of PET imaging of the endocannabinoid system was recently published [23]. Compared to these PET radioligands, [^11^C]PF-06809247 showed similar or higher brain uptake (4 SUV at peak) with clear blocking effect by a defined MAGL inhibitor. This data suggests that [^11^C]PF-06809247 would be a useful PET radioligand for human studies.

One potential limitation of this study is in the use of *K*_*i*_ as the primary outcome measure for determining target occupancy. Theoretically, the rate constant *k*_3_ would be more appropriate, under the conventional assumption that its value is proportional to the available enzyme concentration. However, as shown in Table 2, the estimated *k*_3_ values were highly variable, so this outcome measure was not appropriate for occupancy estimates. This behavior is quite common for irreversibly bound PET radioligands, especially under baseline conditions where enzyme availability is high. Specifically, if baseline *k*_3_ is much higher than *k*_2_ (the clearance rate from tissue), radioligand uptake is flow limited [24]. Thus, using *K*_*i*_ as the outcome measure will likely lead to underestimation of baseline enzyme availability. Under blocking conditions, *k*_3_ is reduced and enzyme availability becomes the rate limiting step, in which case *K*_*i*_ will be more reflective of remaining enzyme availability, i.e., no longer flow limited. It is thus possible that occupancy was underestimated (since baseline *K*_*i*_ is underestimated), in which case the EC_50_ may be overestimated.

## Conclusions

The NHP brain occupancy measured with [^11^C]PF-06809247 showed concentration dependency following dosing of a selective MAGL inhibitor. These data suggest that [^11^C]PF-06809247 can accurately estimate the MAGL. The effective dose of [^11^C]PF-06809247 was similar to other reported carbon-11 PET radioligands. Therefore, [^11^C]PF-06809247 is a promising PET ligand for estimating MAGL and its occupancy in human brain.

## Supplementary Information

**Additional file 1:** Radioligand synthesis.

## Data Availability

The datasets used and/or analysed during the current study are available from the corresponding author on reasonable request.
