# Supplementary material for: Target occupancy study and whole-body dosimetry with a MAGL PET ligand [11C]PF-06809247 in non-human primates
Source: EJNMMI Res. 2022 Mar 4;12:13. doi: 10.1186/s13550-022-00882-2 (PMC8897535; doi:10.1186/s13550-022-00882-2)

**Supplemental text**

***Radioligand synthesis***

^11^C-Methane (^11^C-CH_4_) was produced in-target via the 14 N(p,α) ^11^C reaction on nitrogen mixed with 10% of hydrogen, with 16.4 MeV protons using a GEMS PET trace cyclotron (GE, Uppsala, Sweden). Typically the target gas was irradiated for 15–20 min with a beam current of 35 μA. ^11^C-Labeled methyl iodide, ^11^C-CH_3_I, was produced following the previously published method (1). In short, the produced ^11^C-CH_4_ was released from the target and collected in a Porapak Q trap cooled in liquid nitrogen. After collection, the ^11^C-CH_4_ was released from the trap by heating with flow of helium and subsequently ^11^C-CH_4_ was mixed with iodine vapor from of iodine crystals at 60 °C followed by a free radical reaction at 720 °C. The formed ^11^C-CH_3_I was collected in a porapak Q trap at room temperature and the unreacted ^11^C-CH_4_ was recirculated for 3 min. ^11^C-CH_3_I was released from the Porapak Q trap by heating the trap using a custom-made oven at 180 °C. ^11^C-CH_3_OTf was produced by online transfer of ^11^C-CH_3_I through a glass column packed with silver triflate at 165 °C.

Carbon-11 labeled ^11^C-PF-06809247 was obtained by trapping ^11^C-CH_3_OTf at room temperature in a reaction vessel containing the desmethyl precursor PF-06819821, (0.5 – 1 mg, 1.2 µmol-2.4 µmol) and Cs_2_CO_3_ (10mg, 30 μmol) in dimethylformamide (DMF) (300-400 μL). After end of trapping, deprotection was performed by adding trifluoroacetic acid (TFA) with heating at 100°C for 5 minutes. The reaction mixture was diluted with sterile water (500 μL) before injecting to the built-in high performance liquid chromatography (HPLC) system for the purification of the desired radiolabeled product. The HPLC system consisted of a semi-preparative reverse phase (RP) ACE column (C18, 10 × 250 mm, 5 μm particle size) and a Merck Hitachi UV detector (λ = 254 nm) (VWR, International, Stockholm, Sweden) in series with a GM-tube (Carroll-Ramsey, Berkley, CA, USA) used for radioactivity detection. Acetonitrile / 0.1% Trifluoroacetic acid (TFA), 50:50 (v/v) was used as HPLC mobile phase with a flow rate of 6 mL/min. The radioactive fraction corresponding to pure ^11^C-PF-06809247 was collected and evaporated to dryness. The final purified ^11^C-PF-06809247 was formulated in 6 mL phosphate buffered saline; PBS (pH7.4) and found to be stable after 1 hour. The formulated product was then sterile filtered through a Millipore Millex® GV filter unit (0.22 μm) for further use in vivo. The incorporation yield of ^11^C-CH_3_I was >50% yielding >1000 MBq of the final product. Radiochemical purity was >98% at the time of injection. ^11^C-PF-06809247 was found to be stable in the formulated solution up to 90 min after the end of synthesis. The total synthesis time including purification was 40 min.

1. Andersson J, Truong P, Halldin C. In-target produced [11C]methane: Increased specific radioactivity. Appl Radiat Isot. 2009;67(1):106–10.

**The scheme of radiosynthesis of ^11^C-PF-06809247**

**Figure S1**

**Time activity curves of [^11^C]PF-06809247 in the NHP brain for putamen and frontal cortex at baseline and following pretreatment with a selective MAGL inhibitor (NHP4; 0.42 mg/kg)**


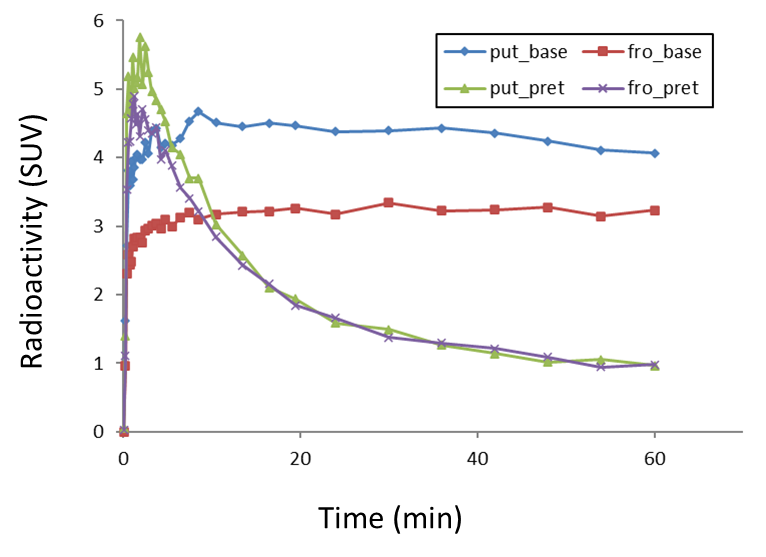


**Figure S2**

**Time-course of plasma concentration of PF-06807893**

**
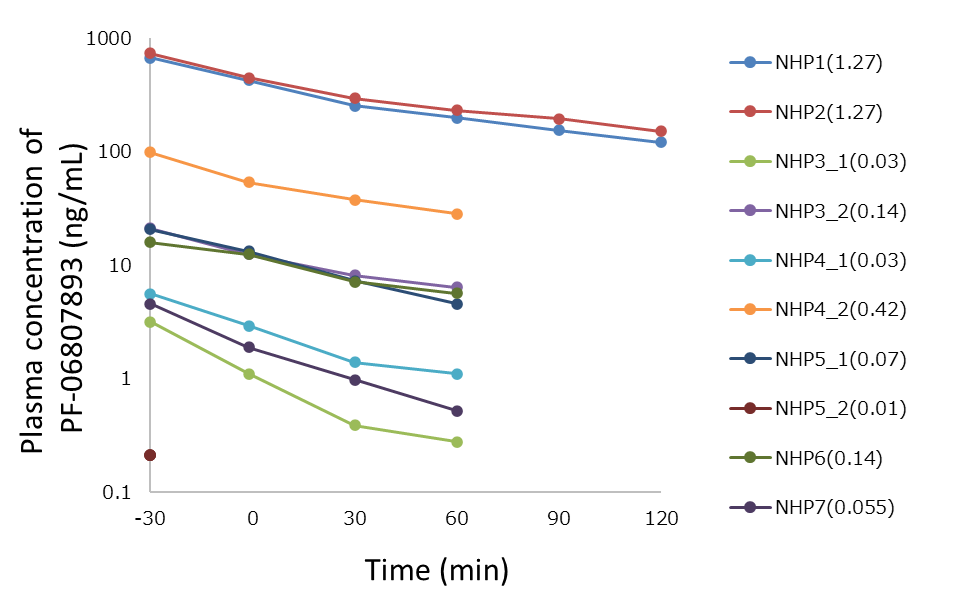
**

**Figure S3**

**Representative plasma input functions at baseline and pretreatment conditions (NHP4; 0.42 mg/kg)**

**
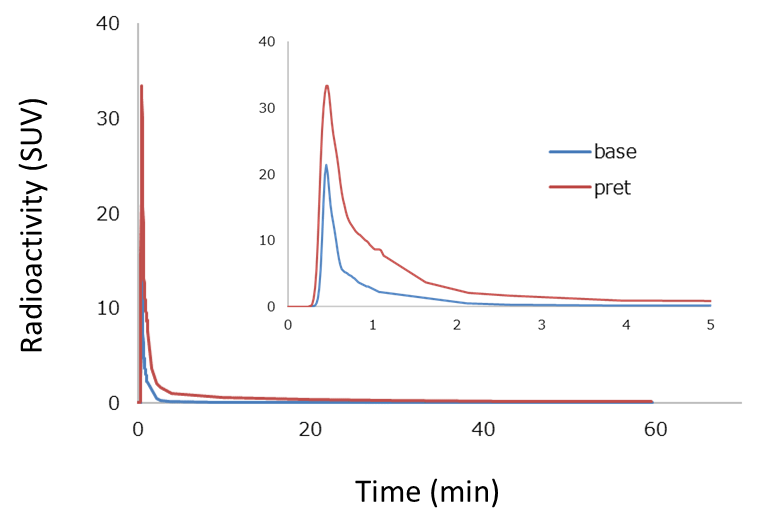
**

**Figure S4**

**The ratio of brain (putamen) uptake to metabolite-corrected plasma on average (N=10) at baseline condition**


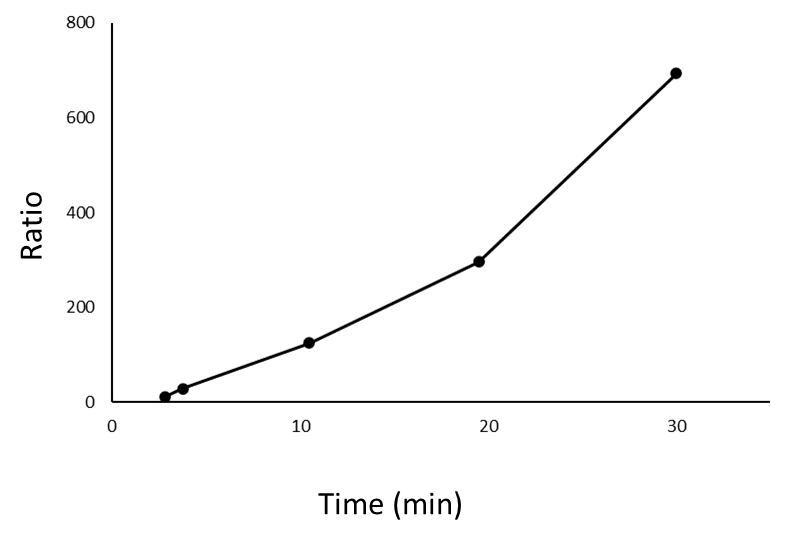


**Figure S5**

**Representative Patlak slope at baseline in the frontal cortex (NHP4; 0.42 mg/kg)**


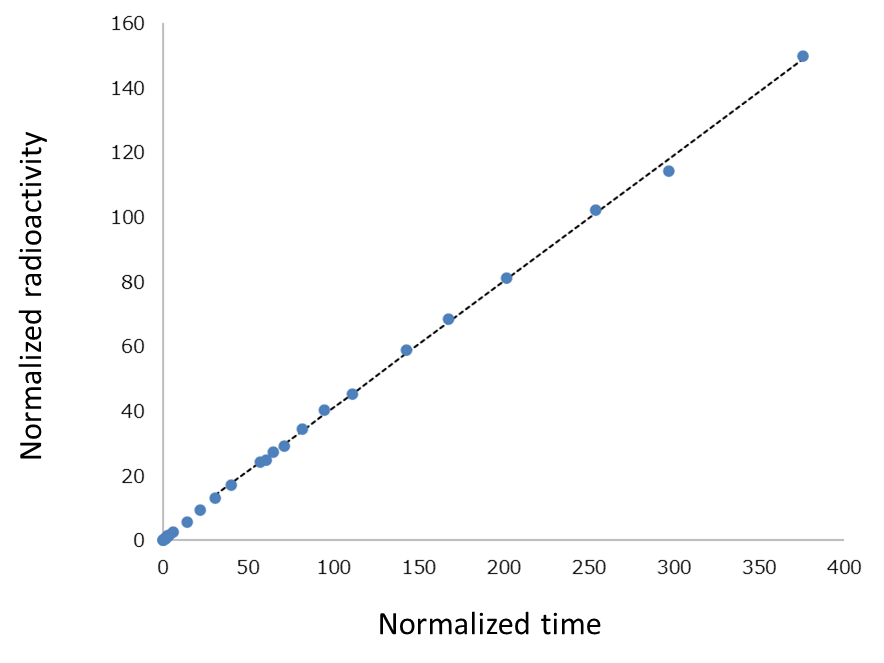

Supplement: Supplementary file 1 — Additional file 1: Radioligand synthesis. [file 13550_2022_882_MOESM1_ESM.docx]
